# Optimizing ex vivo culture conditions to study human gut microbiome

**DOI:** 10.1038/s43705-023-00245-5

**Published:** 2023-04-25

**Authors:** Xin Tao, Wenjin Huang, Lingyun Pan, Lili Sheng, Yuan Qin, Luo Chen, Linhuan Yu, Gaosong Wu, Jianbo Wan, Houkai Li

**Affiliations:** 1 School of Pharmacy, Shanghai University of Traditional Chinese Medicine, Shanghai, China; 2 Institute of Chinese Medical Sciences, University of Macau, Macau, China; 3 Experiment Center for Science and Technology, Shanghai University of Traditional Chinese Medicine, Shanghai, China; 4 College of Acupuncture and Massage, Shanghai University of Traditional Chinese Medicine, Shanghai, China; 5 Institute of Interdisciplinary Integrative Medicine Research, Shanghai University of Traditional Chinese Medicine, Shanghai, China

**Keywords:** Microbiome, Bacteriology

## Abstract

The inter-individual variations of gut microbiome contribute to the different responses toward drug therapy among populations, developing a reliable ex vivo culture method for mixed bacteria is the urgent need for predicting personal reaction to drug therapy. Unfortunately, very few attentions have been paid to the bias that could be introduced during the culture process for mixed bacteria. Here we systemically evaluated the factors that may affect the outcomes of cultured bacteria from human feces. We demonstrated that inter-individual difference of host gut microbiome was the main factor affecting the outcomes of cultured bacteria, followed by the culture medium and time point. We further optimized a new medium termed GB based on our established multi-dimensional evaluation method, which could mimic the status of in situ host gut microbiome to the highest extent. Finally, we assessed the inter-individual metabolism by host gut microbiome from 10 donors on three frequently used clinical drugs (aspirin, levodopa and doxifluridine) based on the optimized GB medium. Our results revealed obvious variation in drug metabolism by microbiome from different donors, especially levodopa and doxifluridine. This work suggested the optimized culture medium had the potential for exploring the inter-individual impacts of host gut microbiome on drug metabolism.

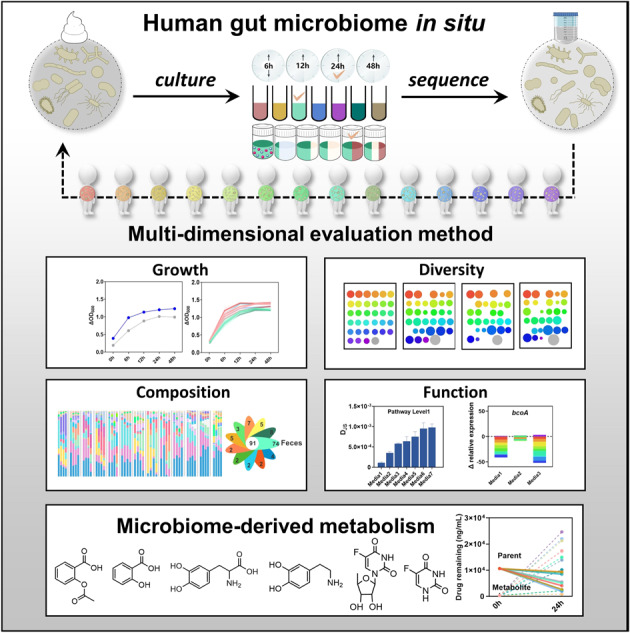

## Introduction

Gut microbiota plays crucial roles in diseases progression and drug metabolism [1–3]. The latest research shows that more than 2/3 chemical drugs could be metabolized to new compounds by at least one human intestinal bacterium [4]. Recent study also indicated the bioaccumulation of therapeutic drugs by human gut bacteria [5]. Thus, the inter-individual difference of gut microbiota is important for determining drug efficacy or side effects in clinic, which has been largely overlooked [1, 6]. Given the individual variation of gut microbiome and narrow therapeutic window of many clinical drugs, personalized medicine is highly valued for improving safety and minimizing toxicity of clinical drugs in recent years [2, 7, 8]. Meanwhile, pharmacomicrobiomics also emphasizes the importance of pre-dose compositional and functional variations of gut microbiome on drug action, fate and toxicity [9–11]. However, the exact microbial role on drug metabolism remains largely unknown due to the complexity of gut microbiome and lack of reliable culture method for mixed bacteria from human population.

The culture-based evidence is crucial for predicting the behavior and outcome of drugs in gastrointestinal tract before absorption. Pilot trial has been practiced by applying high-throughput anaerobic culture techniques in human gut microbiota [12]. Even though not completely reproducing the genuine human gut microbiome, advances in culture conditions make progress in culturing a substantial proportion of intestinal bacteria simultaneously [13, 14]. Previously, microbiota-mediated drug metabolism was trialed based on various culture media and optimized methods [15]. Since most of the human gut microbiota are anaerobic and has complicated interaction, ex vivo culture on human-derived gut bacteria is still a challenging task. Therefore, optimizing the culture conditions for human gut microbiome and developing a multi-dimensional evaluation method is an urgent need for exploring the role of gut microbiome on drug metabolism.

In our current study, we compared the outcomes among a series of culture media that were frequently used for mixed bacteria culture, and developed a multi-dimensional evaluation method for human-derived fecal bacteria culture including the culture time point, bacteria growth rate, diversity (α & β) and similarity in composition and function of gut microbiota. Then, the interpersonal impacts on metabolism of representative clinical drugs (aspirin, levodopa and doxifluridine) were tested based on the optimized human gut microbiota in situ culture method. Our results showed obvious inter-individual difference in drug metabolism by the gut microbiota from ten donors, especially on levodopa and doxifluridine metabolism. These results suggested that the optimized culture method was suitable for exploring the individual impacts of gut microbiota on drug metabolism.

## Material and methods

### Collection and processing of fecal samples from healthy volunteers

The study was approved by the Ethics Committee of Shanghai Hudong hospital in China (Approval number: 2022SHHDKY0602), the written informed consent was given by all volunteers. Fourteen healthy volunteers (5 males and 9 females, age range 22–28 years old) who declared no gastrointestinal pathologies or antibiotics intake at least 2 months prior to stool collection, were recruited to donate the fecal samples employed in this study. Freshly collected feces was brought into anaerobic chamber (90%N_2_, 5%CO_2_, 5%H_2_). One gram of the sample was suspended in 15 mL of sterile phosphate buffer supplemented with 0.1% L-cysteine (PBSc) in a 50 mL sterile falcon tube. The suspension was left standing still for 5 min to let insoluble particles settle, and collected the supernatant to culture quickly [15]. The rest feces were stored at −80 °C.

### ex vivo culture

A small aliquot (500 μL) supernatant was used to inoculate 50 mL different media (Table S1, Table S2), including modified Gifu Anaerobic Medium (mGAM, preparation according to composition from HyServe, Germany), Bryant and Burkey Medium (BB), modified GAM broth, (mGAM_c, commercial product from Hopebio, China), Gifu Anaerobic Medium (GAM), Brain Heart Infusion (BHI), Tryptone Yeast Glucose medium (TYG) [15], and cultures were incubated at 37 °C in an anaerobic chamber. One mL was harvested from each culture at 6, 12, 24 and 48 h used to measure OD_600_ and centrifuged (10,000 *rpm*,1 min) to measure OD_600_ of supernatant, then calculated the ΔOD_600_ and recovered the resulting bacterial pellets to extract DNA and 16 S rRNA gene sequencing.

### qPCR

Total fecal DNA was isolated using a TIANamp Stool DNA Kit. Real-time qPCR was performed using SYBR Green, 96-well plates and the CFX connect Real-Time System. Each well was loaded with a total of 20 µL containing 2 μL DNA, 0.5 μL target-specific primers, 7.5 μL water and 10 μL SYBR Master Mix. Real-time qPCR was performed for 40 cycles, with each cycle consisting of denaturation for 15 s at 94 °C, annealing for 30 s at 60 °C and elongation for 30 s at 72 °C [16]. Relative abundance was quantified using the 2^CT(16S)-CT(target gene)^ [17], and calculated the fold change (the relative abundance of cultured bacteria/ fecal bacteria) for corresponding gene, computed in base 2. The primers used are shown in Supplementary Table S3.

### 16S rRNA gene sequencing

Total DNA was extracted from bacterial pellets using the E.Z.N.A.^®^ soil DNA Kit (Omega Bio-tek, Norcross, GA, U.S.).Bacterial 16 S rRNA gene fragments (V3-V4) were amplified from the extracted DNA using primers 338 F (5′-ACTCCTACGGGAGGCAGCAG-3′) and 806 R(5′-GGACTACHVGGGTWTCTAAT-3′), PCR conditions (30 s at 95 °C, 30 s at 55 °C, and 45 s at 72 °C for 27 cycles), and sequenced on the Illumina MiSeq sequencing platform using PE300 chemical at Majorbio Bio-Pharm Technology Co. Ltd. (Shanghai, China) according to the standard protocols. The raw reads were deposited into the NCBI Sequence Read Archive (SRA) database (PRJNA919413, PRJNA921736, PRJNA921743, PRJNA921822). After demultiplexing, the resulting sequences were merged with FLASH (v1.2.11) [18] and quality filtered with fastp (0.19.6). Then the high-quality sequences were de-noised using DADA2 [19] plugin in the Qiime2 [20] (version 2020.2) pipeline with recommended parameters, which obtains single nucleotide resolution based on error profiles within samples. DADA2 denoised sequences are usually called amplicon sequence variants (ASVs). Taxonomic assignment of ASVs was performed using the Naive bayes consensus taxonomy classifier implemented in Qiime2 and the SILVA 16 S rRNA database (v138). Analyses of the 16 S rRNA microbiome sequencing data was performed using the free online platform of Majorbio Cloud Platform (cloud.majorbio.com).

### Test on drug metabolism by human gut microbiota

In an anaerobic chamber, 200 μL D1-D10 glycerol stock was cultured in 10 mL GB respectively. Cultures were grown for 24 h at 37 °C in an anaerobic chamber. After 24 h, 400 μL cultures and 4 mL of each drug (aspirin, levodopa and doxifluridine) were add to 36 mL GB (with each drug having a concentration of 500 μM in GB). In addition, 4 mL of each drug was also incubated similarly in a no-microbiome, GB control. The no-microbiome control distinguishes cases of passive drug degradation. Experiments and controls were allowed to incubate under the same conditions for 6 h, 12 h and 24 h. After incubation, cultures were centrifuged (10,000 *rpm*,1 min), the supernatant was collected and frozen at −80 °C until use [15].

### Sample preparation and analysis

The supernatant samples (100 μL) were deproteinized with acetonitrile (900 μL) containing 0.9 μg/mL internal standard (benzoic acid for aspirin and salicylic acid, methyldopa for levodopa and dopamine, 5-Chlorouracil for doxifluridine and 5-Fluorouracil) [4]. The sample was vigorously vortex-mixed, and then centrifuged at 12,000 *rpm* for 20 min. The resulting supernatant was transferred to LC vial and a 1 μL aliquot was injected into the LC-MS/MS analysis system. Calibration curves were generated by plotting the peak area ratio of the analyte to IS versus the concentration of the analyte, using least-square linear regression. The correlation coefficients of the calibration curves were >0.99. The calibration curve equations were y = 347.54x + 0.4552 (aspirin), y = 8.3759x − 7.9087 (salicylic acid), y = 1126.8x + 0.3252 (levodopa), y = 299.28x + 6.4806 (dopamine), y = 0.5103x + 0.0049 (doxifluridine), y = 0.1328x + 0.0034 (5-Fluorouracil).

### LC-MS/MS analysis

The LC-MS/MS system consisted of an ExionLC100 with a Triple-Quad 5500 (AB SCIEX, USA) equipped with an electrospray ionization (ESI) source and Analyst 1.6.3 software for data acquisition and analysis. Chromatographic separation was achieved with a ACQUITY UPLC HSS T3 (2.1 × 100 mm, 1.8 μm) and oven temperature maintained at 40 °C. For detection of aspirin, salicylic acid, levodopa and dopamine, the mobile phase consisted of 0.1% formic acid-water (solvent A) and acetonitrile (solvent B), the following gradient elution was used at a flow rate 0.2 mL/min: 0–2 min: 90% A, 2–2.1 min: 70% A, 2.1–3 min: 30% A, 3–5 min: 10% A, 5–7 min: 5% A, 7–8 min, 90% A. Sample injection volume was 1 μL. With the chromatography conditions described above, aspirin and salicylic acid were eluted out at 4.33 min and 4.53 min, benzoic acid was eluted out at 4.40 min; levodopa and dopamine were eluted out at 1.40 min and 1.38 min, methyldopa was eluted out at 1.56 min. For detection of doxifluridine, 5-Fluorouracil and 5-Chlorouracil, the mobile phase consisted of a mixture of 5 mM ammonium formate with 0.025% acetic acid solution (solvent A, pH = 3.8) and methanol (solvent B), the following gradient elution was used at a flow rate 0.3 mL/min: 0–2 min: 90% A, 2–3.8 min: 60% A, 3.8–4.8 min: 10% A, 4.8–5.5 min: 90% A. Sample injection volume was 1 μL. With the chromatography conditions described above, doxifluridine and 5-Fluorouracil were eluted out at 3.59 min and 1.28 min, 5-Chlorouracil was eluted out at 1.83 min.

After chromatographic separation, the eluent was introduced to the mass spectrometer via an electrospray ionization (ESI) ion source. The mass spectrometer was conducted in the negative ionization mode for the detection of aspirin, salicylic acid, doxifluridine and 5-Fluorouracil, but in the positive ionization mode for the detection of levodopa and dopamine. The MS detector was operated in multiple reaction monitoring (MRM) mode. The optimized mass spectrometric parameters, Q1 and Q3 fragmention, collision energies (CE), declustering potential (DP), entrance potential (EP), collision cell exit potential (CXP) descriptions are shown in Table S4 [21–25].

Nitrogen was used as nebulizing, curtain and collision gases. For detection of aspirin, salicylic acid, benzoic acid, levodopa, dopamine and methyldopa, the curtain gas (CUR) was 35 psi; the ion source gas (GS1) and the ion source gas 2 (GS2) were 55 psi and 55 psi; the source temperature (TEM) and ionspray voltage floating (ISVF) were 550 °C and 5500 V. For detection of doxifluridine, 5-Fluorouracil and 5-Chlorouracil, CUR was 25 psi; the GS1 and the GS2 were 50 psi and 50 psi; the TEM and ISVF were 550 °C and −4500V. Other MS parameters were adopted from the recommended values for the instrument.

### Statistical analysis

Data are shown as means ± SD. All the bar plots in this study were generated with Prism 8.0 (GraphPad, La Jolla, CA, USA). Differences between two groups were calculated by *T*-test, *p* < 0.05 was considered statistically significant.

## Results

### Evaluating the impacts of culture time on outcomes of human fecal bacteria culture

**Fig. 1:**
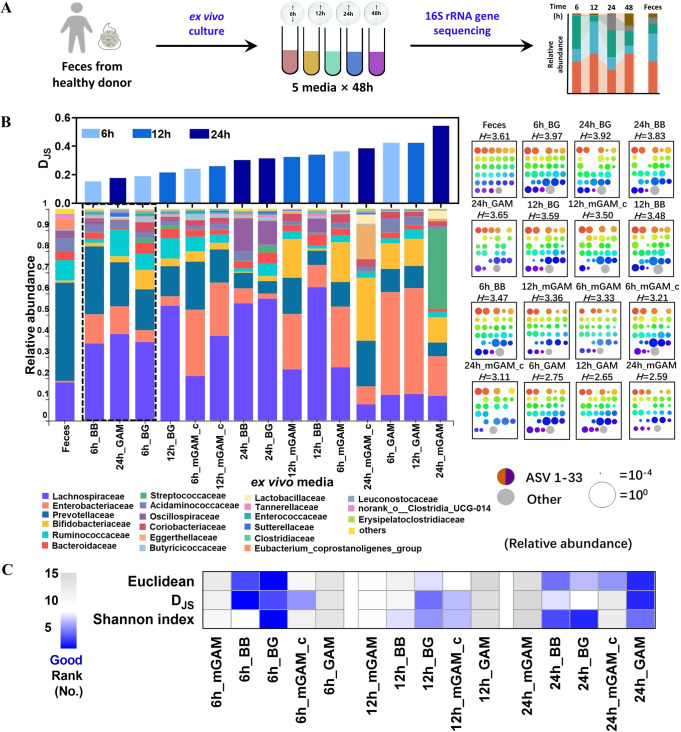
Investigation of culture time for human-fecal samples. **A** Flow chart of the experiment **B** Family level bacterial composition of the sample (left), including original fecal sample and its ex vivo cultures, grown anaerobically in 5 different media at incubation time of 6,12, and 24 h. Cultures are ordered according to their Jensen-Shannon divergence (D_JS_) from the original fecal sample (upper axes, computed at the family level). ASV level bacterial composition of the sample (right), where each square represents one sample. Rainbow colored dots represent the relative abundance of individual ASVs. Samples are ordered by their Shannon index at the ASV level, computed in base 2 and shown above each square. **C** Comprehensive rank of 15 cultures, which are ordered according to the Euclidean (ASV level) and D_JS_ (family level) from the original fecal sample (rank from 1 to 15 in ascending order), and the Shannon index (rank from 1 to 15 in descending order), the smaller rank indicates higher similarity and diversity.

Culturing time and medium are two critical factors in affecting the outcomes of bacteria culture. To determine the optimal incubation time, freshly collected feces from one health donor were cultured in five different media that were frequently used for culture of mixed bacteria [15, 26, 27] and sampled at 6, 12, 24, and 48 h (Fig. 1A). Based on the growth curve, all five cultures reached stationary phase within 12 h, while mGAM_c and GAM showed higher bacterial density (Fig. S1A). Besides, the bacteria abundance in each medium at the phylum level was detected by qPCR and compared with their corresponding fresh feces [17]. The results showed that the abundance of Firmicutes was increased, whereas Bacteroidetes and Verrucomicrobia was decreased along with the extension of culture time, especially at 48 h. Meanwhile, the microbial functions were assayed by evaluating the expression of some representative genes involved in bile acids metabolism and SCFAs production including *baiJ*, *bsh*, and *bcoA* between cultured bacteria in various media and their fresh feces. The most significant variations took place at 48 h in each culture medium compared with their fresh feces (Fig. S1B, C). These data suggested that 48 h was not appropriate for mixed bacteria culture.

Next, the bacterial diversity among cultured samples after 6 h, 12 h and 24 h was evaluated by using 16 S rRNA gene sequencing. First of all, the similarity between the cultures in various media and fresh feces was quantified by using the Jensen-Shannon divergence (D_JS_) and Euclidean at the family level [15, 28, 29]. Lower value of D_JS_ or Euclidean suggests the more similar to the bacteria in fresh feces. We observed BB culture medium at 6 h, GAM culture medium at 24 h, and BG culture medium at 6 h showed the lowest D_JS_ divergence from fresh feces (Fig. 1B, C). At the single ASV level, BG culture medium at 6 h, BG culture medium at 24 h, and BB culture medium at 24 h captured the majority of the bacterial diversity in fresh feces. In addition, we ranked the 15 tested culture conditions based on the similarity of composition, in which blue color means more similar to bacteria in fresh feces. It was obvious that cultures in different at 6 h and 24 h were closer to the bacteria of their fresh feces based on values of D_JS_, Euclidean or Shannon index (Fig. 1B, C). Therefore, 6 h and 24 h of culture were used for the subsequent optimization process.

### Optimizing the ex vivo culturing methodology for human fecal bacteria

**Fig. 2:**
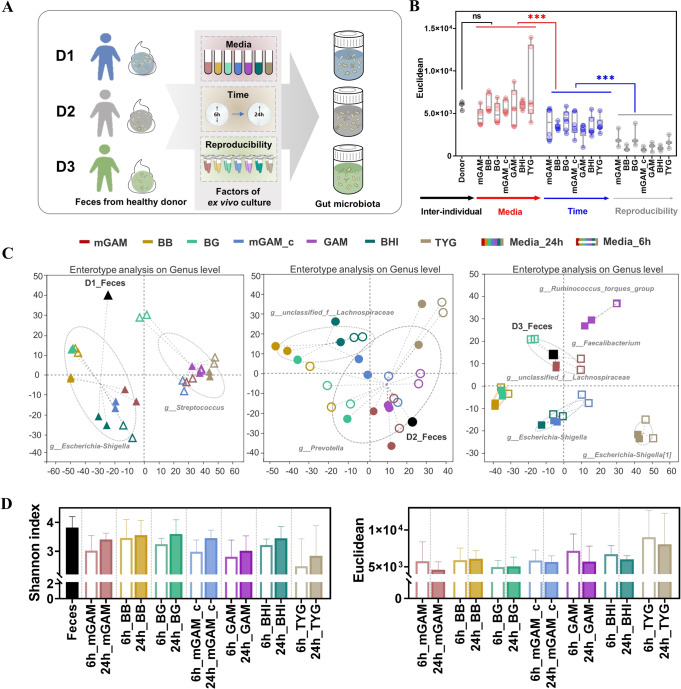
Evaluating the ex vivo culturing system for human-fecal samples. **A** Flow chart of the experiment **B**. Euclidean of factors at the ASV level **C** Enterotype of fecal sample and its cultures. **D** Shannon index and Euclidean at the ASV level.

To develop an optimized ex vivo culture method that could maximally reflect the genuine status of intestinal bacteria, a systemic methodology study was performed by including seven types of culture media on feces from three different donors and two culture time points (6 h and 24 h) (Fig. 2A) [15]. The results showed that the inter-individual differences among three donor samples and types of media were the main factors affecting the outcomes of culture, while culture time or sample duplicates exhibited minor impacts on outcomes of bacteria culture (Fig. 2B).

To further explore the influence of different culture media on gut microbiota composition, enterotype was analyzed among samples from three different donors by different culture media and time points. The enterotype of fresh feces from 3 donors was different, but shared similar enterotypes with their cultures, respectively (Fig. S2). As shown in Fig. 2C, fresh feces, mGAM culture medium at 24 h, BG culture medium at 24 h, mGAM_c culture medium at 24 h, BB and BHI at both time points clustered into *g_Escherichia-Shigella*, while mGAM culture medium at 6 h, BG culture medium at 6 h, mGAM_c culture medium at 6 h, GAM and TYG at both time points clustered into *g_Streptococcus* among donor one. Notably, cultures from the same medium but different time points were clustered into the same enterotype including BB, GAM, BHI and TYG, suggesting the impact of culture medium was greater than culture time. In donor two, fresh feces, mGAM, mGAM_c, GAM and TYG at two time points clustered into *g_Prevotella*. In donor three, 5 different enterotypes appeared based on different culture media after 6 h or 24 h culture, while the 2 time points cultures in BG medium were clustered with BB or mGAM (Fig. 2C). Overall, these data implied that the most significant factor for affecting gut microbiota composition and diversity was the inter-individual differences from different donors, and followed by the types of culture medium. In agreement with the above observations, the following PERMANOVA analysis using multivariate models [30] also showed that inter-individual differences among donors accounted for 37.5% variation, while the media and time points accounted for 31.8% and 3.4% variations respectively (Table 1). In addition, the Shannon index and Euclidean distance consistently suggested that the outcomes of 24 h culture in different media were relatively closer to fresh feces than 6 h (Fig. 2D).

**Table 1:** PERMANOVA analysis of factors (Bray_curtis).

Factor	Df	SumsOfSqs	MeanSqs	F.Models	*R*^2^	Pr (>F)
Inter-individual	2	5.830	2.915	50.256	0.375	0.001
Media	6	4.945	0.824	14.212	0.318	0.001
Time	1	0.522	0.522	8.995	0.034	0.001
Reproducibility	1	0.005	0.005	0.094	0.000	0.999
Residuals	73	4.234	0.058	—	0.273	—
Total	83	15.536	—	—	1	—

**Fig. 3:**
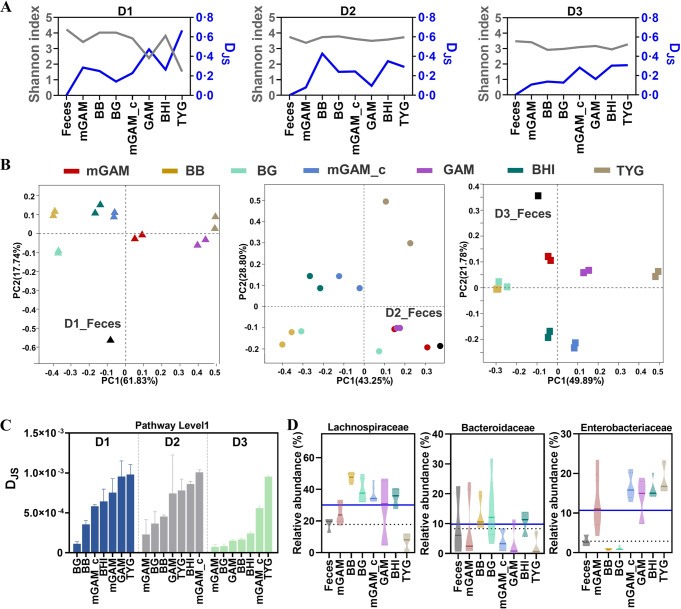
Screening of different culture media. **A** Shannon index at the ASV level and D_JS_ at the family level. **B** Bray curtis based Principal co-ordinates analysis (PCoA) among the samples at genus level. **C** D_JS_ from the fecal sample, computed at the pathway level 1. **D** Abundance of top 3 gut microbiota at the family level (dashed line means the abundance of gut microbiota in fresh feces, solid line means the average abundance of gut microbiota in 7 media).

In addition to enterotype analysis, the comparisons among different culture media at 24 h were further performed based on variations in bacterial diversity and function. Shannon index showed that most of the trialed media yielded similar diversity with their fresh feces from 3 donors, except for the relatively low diversity observed by GAM and TYG in donor one (Fig. 3A). Meanwhile, samples in different culture media showed variation in values of D_JS_ and Euclidean among 3 donors, in which BG medium yielded consistent low values among 3 donors (Fig. 3A, Fig. S3A). We also observed that gut microbiota composition in the cultures of mGAM and BG were similar with their fresh feces by community barplot analysis (Fig. S3B). Principal co-ordinates analysis (PCoA) showed that all the cultures were different with their fresh feces from 3 donors, while samples of mGAM and BG culture were relatively closer to fresh feces than other media (Fig. 3B). Function prediction (pathway level 1) were calculated using the PICRUSt2 to obtain the corresponding functional information and abundance information in each sample. Then the D_JS_ of function (gene family) abundance was calculated between fresh feces and cultures [31, 32]. In agreement with the compositional characters, the functional analysis showed that the cultured bacteria in mGAM, BG and BB media had relatively higher similarity with their fresh feces than other media based on D_JS_ value at the pathway level 1. (Fig. 3C).

Furthermore, the bacterial variations of the top 10 abundance at family level was compared to their fresh feces based on the results of 16 S rRNA gene sequencing. For Lachnospiraceae, its relative abundance was increased after culture in most media, except for decreasing in TYG. For Bacteroidaceae, it showed the trend of increasing after culture in BB, BG and BHI, and decreasing in mGAM, mGAM_c, GAM and TYG. For Enterobacteriaceae, its abundance was increased after culture in the five media of mGAM, mGAM_c, GAM, BHI and TYG, but decreased in BB and BG (Fig. 3D). Similar variations were observed in other bacteria such as Bifidobacteriaceaae, Sutterellaceae and Oscillospiraceae (Fig. S3C). Altogether, these results indicated that different extent of variations were present in any kind of tested media, while mGAM and BG had relatively better performance in gut microbiota composition and function than the rest.

### Culture media modification and validation

Based on the above experiments, further comparison was performed between mGAM and BG. The results showed that bacteria cultured in BG had higher α diversity and similarity to fresh feces than those in mGAM (Fig. S4A–C). Previous study suggested that the overgrowth of Enterobacteriaceae in mGAM could be prevented by mixing with BB to form a new medium BG [15], which imspired us to modify the medium by mixing different media with opposite effect on bacteria growth. Thus, BG was chosen as a basic medium for optimizing an in-house modified medium by including different proportion of mGAM or TYG medium. Given the observed opposite impacts on the abundance of the main families of Lachnospiraceae, Bacteroidaceae and Enterobacteriaceae between BG, mGAM and TYG media (Fig. 3D), a series of mixed media were acquired based on the proportions of these three media (Table S5). Obviously, the mixture of 10–30% of TYG with BG (B9T, B8T, B7T) did not alter the outcome of culture bacteria compare to BG itself either at composition or function (Fig. S4D–F). In contrast, the modified medium with increased proportion of TYG in BG (B3T) produced worse performance in respect to bacterial diversity and function analysis (Fig. S4G–I). Then, two more balanced modified media were designed by using equal percentage of BG or mGAM with or without 30% TYG, named GB or BGT medium respectively (Table S5). Though there was no difference in diversity and similarity with fresh feces among BG, GB and BGT (Fig. S5A–C), the abundance of Lachnospiraceae in GB and BGT was closer to fresh feces compared with BG (Fig. S5D), implying the potential of either GB or BGT medium for mixed bacteria culture.

**Fig. 4:**
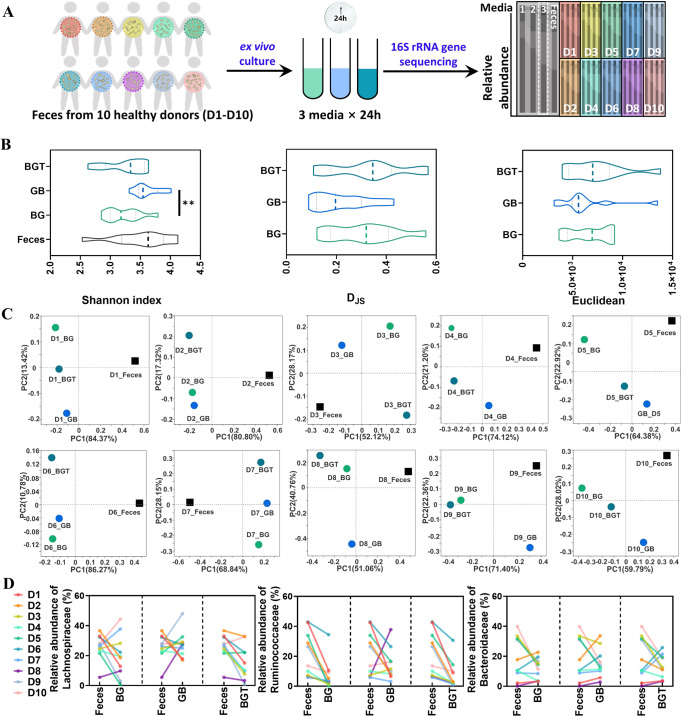
The validation of optimized culture media. **A** Flow chart of the experiment. **B** Shannon index at the ASV level, D_JS_ and Euclidean at the family level. **C** Bray curtis based PCoA in group of BG, GB and BGT. **D** Abundance of top 3 gut microbiota at the family level.

To test the reliability of modified media for human gut microbiota culture, the comparison was performed among BG, GB and BGT by including ten different donor fecal samples (Fig. 4A). The bacteria growth curve showed that bacteria in GB medium grew faster than BG or BGT media for most donor samples (Fig. S6). Next, multivariate PERMANOVA analysis showed inter-individual difference was the most significant contributor shaping the human gut microbiota with about 70.1% explaining power, while the media factor accounted 11.0% (Table 2). In addition, the subsequent multivariate PERMANOVA analysis showed that GB had lower impact on variation of bacteria after culture than BG and BGT (Table S6). In agreement with these observations, GB cultures had more similar Shannon index, as well as higher similarity with fresh feces based on D_JS_ and Euclidean values than either BG or BGT media (Fig. 4B, Fig. S7A). PCoA showed that 5 out of 10 donor samples in GB medium were clustered with fresh feces alongside PC1 with the highest interpretation rate (Fig. 4C), implying that the bacteria in GB culture medium were of high similarity with fresh feces than the rest. In addition, specific analysis on the top 10 class of bacteria at family level after culture showed that the compositional changes were relative stable in GB medium among the 10 donors, especially in Lachnospiraceae, Enterobacteriaceae, Streptococcaceae, Veillonellaceae and Bifidobacteriaceae. (Fig. 4D, Fig. S7B). In addition, the functional analysis indicated that bacteria in GB culture were also closer to fresh feces than the other two media based on the evaluations on expression of genes encoding bile acid and SCFAs metabolism enzyme *bcoA, bsh* and *baiJ*, and D_JS_ value at pathway level 1 (Fig. S7C, D).

**Table 2:** PERMANOVA analysis of factors (Bray_curtis).

Factor	Df	SumsOfSqs	MeanSqs	F.Models	*R*^2^	Pr (>F)
Inter-individual	9	4.149	0.461	7.416	0.701	0.001
Media	2	0.654	0.327	5.261	0.110	0.001
Residuals	18	1.119	0.062	—	0.189	—
Total	29	5.923	—	—	1.000	—

**Fig. 5:**
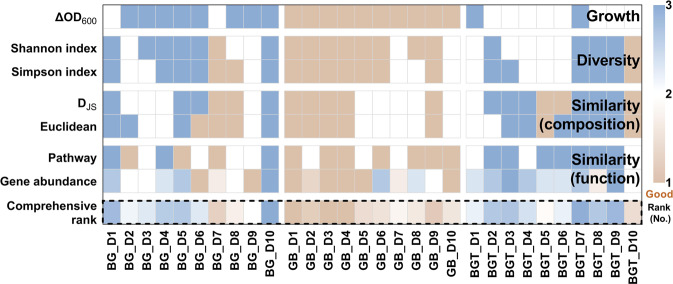
Multi-dimensional evaluation of mixed media. Comprehensive rank of 30 cultures (3 media and 10 donors), which are ordered according to the D_JS_ of composition, Euclidean, D_JS_ of pathway level 1, variation of gene abundance from the original fecal sample (rank from 1 to 3 in ascending order), ΔOD_600_, Shannon index and Simpson index (rank from 1 to 3 in descending order), and calculated their average as comprehensive rank of each culture. The smaller rank indicates higher growth, similarity and diversity.

Taken together, a comprehensive ranking was calculated by including bacteria growth, α and β diversity, and bacterial functions, which indicated that GB medium was superior to the other two media based on the multi-dimensional evaluation (Fig. 5).

### Personalized drug metabolism by human gut microbiota ex vivo

**Fig. 6:**
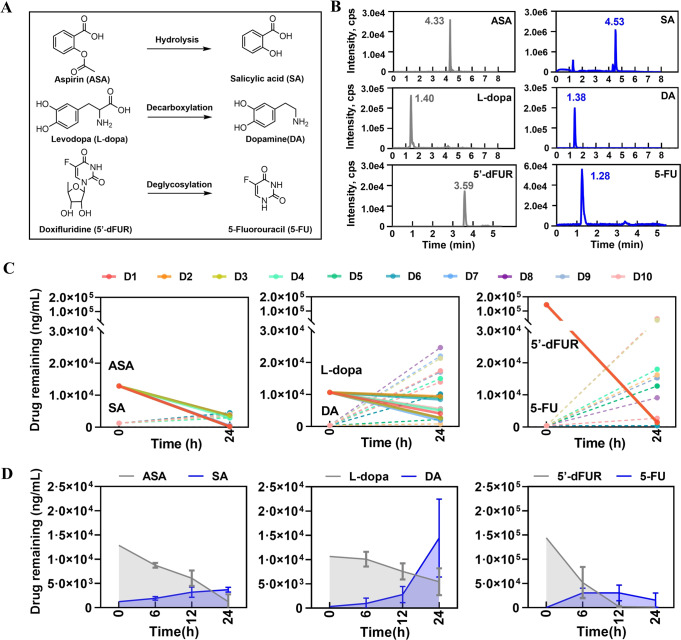
Personalized drug metabolism by human gut microbiota ex vivo. **A** Metabolism of ASA, L-dopa and 5′-dFUR. **B** The chromatograms of ASA, SA, L-dopa, DA, 5′-dFUR and 5-FU. **C** Drug remaining after incubation with gut microbiota on 24 h for each donor-drug combination. **D** Drug remaining after incubation with gut microbiota on 6, 12, and 24 h.

Based on the optimized GB medium for human fecal bacteria culture, we next explored the impacts on drug metabolism by individual gut microbiome from different donors. Three representative drugs were trialed by gut microbiome from ten individual donors including aspirin (ASA), levodopa (L-dopa) and doxifluridine (5′-dFUR) that could be metabolized by microbial enzymes (Fig. 6A, B, Fig. S8) [15, 33, 34]. First of all, the inter-individual differences in microbiota-mediated drug metabolism were evaluated among cultured bacteria from 10 donors. The results showed that the inter-individual differences were not very obvious in the final concentration of parent drugs of ASA and 5′-dFUR, however, high variation was observed in L-dopa. Meanwhile, high inter-individual variations were present in the generation of DA and 5-FU from their parent drugs among the cultured bacteria from 10 different donors, whereas few difference was observed in the generation of SA among 10 donors (Fig. 6C). Next, the amount of parent compound of the 3 drugs decreased time-dependently within 24 h culture, in which ASA and 5′-dFUR were almost nondetectable, but L-dopa was half to its original concentration after 24 h culture. Meanwhile, the quantities of metabolites SA and DA were accumulated and reached the highest point at 24 h, while the concentration of 5-FU was relatively stable during the 24 h observation (Fig. 6D). These results suggested that the varied personal responses to L-dopa or 5′-dFUR due to the metabolism by individual gut microbiome could be captured by this established human gut microbiota ex vivo culture method.

## Discussion

Our current study obtained an optimized GB medium for the ex vivo gut microbiome culture based on a multi-dimensional evaluating strategy including culture time, bacteria growth, α and β diversities, and expression of representative functional genes. We further showed the personalized drug metabolism by using the optimized GB medium for human fecal bacteria.

Though extremely difficult of human gut microbiome culture, anaerobic techniques were increasingly applied to culture human gut microbiota in situ [12] by using single medium like GMM, GAM, mGAM, or the mixed BG medium [12, 15, 35]. However, a huge gap still existed between the cultured bacteria and the real state of intestinal bacteria with any of the existing culture media. In current study, we optimized the culture conditions by integrating a multi-dimensional strategy including bacteria growth, α and β diversity, similarity with fresh feces and reproducibility based on the fecal samples from multiple healthy donors. Except for the observation of microbial composition, we also evaluated the functional status of gut microbiota by the function prediction with PICRUSt2 and the representative genes involved in bile acid metabolism and SCFA production by qPCR, which are driven by gut microbiota and play significant roles in host health [3]. In agreement with the main impactor of inter-individual difference on gut microbiome [15, 35], we proved that the type of culture medium was the key factor for introducing variations to the outcome of bacteria culture, and followed by culture time. Unfortunately, the impacts of culture medium on gut microbiome were largely overlooked because different kinds of culture medium for mixed bacteria culture were used in most studies such mGAM, GAM, GMM, BHI with little attention to the variations that may be introduced by culture medium, as well as the culture time [12, 15, 26, 27, 35–37].

In our current study, we first demonstrated that the impacts of culture time on outcomes of cultured bacteria was minor than medium. Nevertheless, we optimized that 24 h culture was relatively suitable for mixed bacteria culture compared to 6 h, 12 h or 48 h time points because of the higher similarity with their fresh feces from individual donors than other time points based on Shannon index, D_JS_ or Euclidean in different culture media. Actually, the 24 h culture time was widely used for most ex vivo culture [15, 38], which were consistent with our conclusion. Based on the optimized culture time, we further evaluated the outcomes of cultured bacteria by including a series of “well-established” and frequently used media for mixed bacteria culture such as mGAM, GAM, BB, BG, TYG, and BHI. Since BG medium was a previously optimized medium by mixing BB and mGAM in unequal ratio (7:3) [15], we further optimized the formula by mixing equal ratio of BG and mGAM with or without TYG medium, termed as GB or BGT in current study, for the purpose of balancing the huge variations in relative abundance of either Lachnospiraceae or Enterobacteriaceae happened in BG medium. Based on our established multi-dimensional evaluation strategy, we demonstrated that GB medium was superior to any other trialed medium in the context of fecal samples from ten different donors. Notably, the relative abundance of the main bacteria among individuals was maintained well in GB medium at family level including Lachnospiraceae, Ruminococcaceae and Enterobacteriaceae. These results suggested that the optimized GB medium was better than BG and other trialed media for culture of mixed bacteria from human fecal sample.

Inter-individual differences toward drug therapy are very common in clinic, in which personalized gut microbiota-mediated drug metabolism is increasingly recognized in recent years. Previously, Zimmermann et al. revealed that about 2/3 of clinical drugs that could be metabolized by at least one or more gut bacteria [4]. Moreover, some of the drugs are bioaccumulated within gut microbiota, leading to variation in either pharmacodynamics or pharmacokinetics [5]. Therefore, the differences of human gut microbiome contribute greatly to the variable drug responses that are previously assumed to be mainly due to the polymorphism of drug metabolism genes, specifically P450 enzymes in microsome of liver [1]. Based on our developed culture method, we further evaluated the inter-individual differences on drug metabolism by personal gut microbiome. Three clinic drugs, ASA, L-dopa or 5′-dFUR were included for comparison among gut microbiome from ten different donors by using our GB culture medium. The results indicated that all the three drugs were obviously metabolized by gut microbiome that was consistent with previous report [15, 33, 34]. Meanwhile, inter-individual difference of the production of metabolite DA or 5-FU from their parent drug L-dopa or 5′-dFUR was very significant among ten donors, but not in ASA metabolism. These results implied that the optimized GB medium was capable for capturing the inter-individual differences in drug metabolism by personalized gut microbiome through ex vivo culture.

In summary, we systemically evaluated the performance of a series of formulated culture media for mixed bacteria culture from human fecal samples. We then optimized the anaerobic ex vivo culture method by developing a new formulated GB medium and cultured for 24 h for human gut microbiome ex vivo culture and drum metabolism study based on the multi-dimensional parameters including bacterial diversity (α & β), similarity of microbial composition (D_JS_ & Euclidean) and bacterial function (pathway & gene).

## Supplementary information

Supplementary information

## Data Availability

Raw fastq files of this study were deposited to the Sequence Read Archive database (PRJNA919413, PRJNA921736, PRJNA921743, PRJNA921822).
