# Supplementary material for: Optimizing ex vivo culture conditions to study human gut microbiome
Source: ISME Commun. 2023 Apr 25;3:38. doi: 10.1038/s43705-023-00245-5 (PMC10130157; doi:10.1038/s43705-023-00245-5)
Supplement: Supplementary file 1 — Supplementary information [file 43705_2023_245_MOESM1_ESM.docx]

Supplementary Information

**Optimizing *ex vivo* culture conditions to study human gut microbiome**

Xin Tao^1,2^, Wenjin Huang^1^, Lingyun Pan^3^, Lili Sheng^1^, Yuan Qin^4^, Luo Chen^1^, Linhuan Yu^1^, Gaosong Wu^5^ and Jianbo Wan^2^, Houkai Li^1^*

^1^ School of Pharmacy, Shanghai University of Traditional Chinese Medicine, Shanghai, China

^2^ Institute of Chinese Medical Sciences, University of Macau, Macau, China

^3^ Experiment Center for Science and Technology, Shanghai University of Traditional Chinese Medicine, Shanghai, China

^4^ College of Acupuncture and Massage, Shanghai University of Traditional Chinese Medicine, Shanghai, China

^5^ Institute of Interdisciplinary Integrative Medicine Research, Shanghai University of Traditional Chinese Medicine, Shanghai, China

**Corresponding author:**

Houkai Li

School of Pharmacy, Shanghai University of Traditional Chinese Medicine, Shanghai, China

Phone:86-21-51322729

Email:[hk_li@shutcm.edu.cn](mailto:hk_li@shutcm.edu.cn)

**Keywords:** *ex vivo* culture, gut microbiome, drug metabolism, 16S rRNA gene sequencing

**Contents:**

**Table S1.** The chemicals, media, and reagents…………………………………..…….2

**Table S2.** The composition of 7 culture media……………………………………….….3

**Table S3.** The primers used in qPCR…………………………………………………….4

**Table S4.** MRM transitions and the optimum LC–MS/MS conditions…………………5

**Table S5.** The composition of 6 mixed media……………………………………………6

**Table S6.** PERMANOVA analysis of factors (Bray_curtis)………………………………7

**Table S7.** Amount of sequence data for all the samples (Reads per sample)………8

**Fig. S1** Investigation of culture time for human-fecal samples…………………….10

**Fig. S2** Enterotype of fecal samples and their cultures……………………………..11

**Fig. S3** Composition of gut microbiota with high abundance……………………….12

**Fig. S4** The modification of culture media……………………………………………..13

**Fig. S5** The optimization of culture media……………………………………………..14

**Fig. S6** Growth condition of optimized culture media………………………………..15

**Fig. S7** Comparing of optimized culture media………………………………………..16

**Fig. S8.** The mass spectrum……………………………………………………………..17

**Table S1. The chemicals, media, and reagents**

| Reagent or resource | Source | Identifier |
| --- | --- | --- |
| Chemicals, media and reagents | | |
| Brain heart infusion | BD | 237500 |
| Casein enzymic hydrolysate | Sangon | A10085-0100 |
| Dextrose | Sangon | A610291 |
| Digested serum | YuanMu | YR0305 |
| GAM broth, modified | Hopebio | HB8518 |
| Hemln | Sigma-Aldrich | H9039-1G |
| KH_2_PO_4_ | Sinopharm | 10017618 |
| L-Arginine | Sangon | A600205-0100 |
| L-Cysteine hydrochlorlde | Sinopharm | XW00528911 |
| Liver extract | Hongrun Baoshun | Y037 |
| L-Tryptopan | Sinopharm | 73-22-3 |
| Beef extract | Sangon | A600114-0100 |
| NaCl | Sinopharm | 10019318 |
| Peptone | Sangon | A100636-0100 |
| Proteose peptone | Sangon | A600241-0100 |
| Resazurin | Sangon | A606726-0001 |
| Sodium acetate | Sigma-Aldrich | S8750-250G |
| Soya peptone | Sangon | A600214-0100 |
| Soluble starch | Sangon | A500904-0500 |
| Sodium thioglycolate | Sangon | A610265-0025 |
| Tryptone | BD-AB | 211705 |
| Vitamin K1 | Sangon | A606528-0005 |
| Yeast extract | BD-AB | 212750 |
| PBS(10×) | Beyotime | ST476 |
| L-Cysteine | Vetec | V900400-100G |
| Hieff®qPCR SYBR Green Master Mix | Yeasen | 11201ES03 |
| TIANamp Stool DNA Kit | TIANGEN | DP328-02 |
| Drugs | | |
| Aspirin | Sigma | A2093-100G |
| Salicylic acid | Solarbio | SS8860 |
| Benzoic acid | Macklin | B803151 |
| Levodopa | Sigma | D9628-5G |
| Dopamine | Macklin | A849306 |
| Methyldopa | Solarbio | IM2680 |
| Doxifluridine | Sigma | F8791-100MG |
| 5-Fluorouracil | Sigma | F6627-1G |
| 5-Chlorouracil | YuanYe | B24730 |

**Table S2. The composition of 7 culture media**

| Composition Name | Concentration ( g/L ) | | | | | | |
| --- | --- | --- | --- | --- | --- | --- | --- |
|  | mGAM | BB | BG | mGAM_c | GAM | BHI | TYG |
| Dextrose | 0.50 | / | 0.15 | 3.00 | 3.00 | 0.16 | 2.00 |
| Soluble Starch | 5.00 |  | 1.50 | 0.30 | 5.00 | / | / |
| Calf Brains | / |  | / | / | / | 15.76 |  |
| Beef Hearts |  |  |  |  |  | 19.70 |  |
| Yeast Extract | 2.50 | 5.00 | 4.25 | 5.00 | 5.00 | / | 5.00 |
| Meat Extract | 2.20 | 7.50 | 5.91 | 2.00 | 2.20 |  | / |
| Liver Extract | 1.20 | / | 0.36 | 1.20 | 1.20 |  |  |
| Digested Serum | 10.00 |  | 3.00 | 13.50 | 13.50 |  |  |
| Peptone | 5.00 |  | 1.50 | / | 10.00 |  |  |
| Soya peptone | 3.00 |  | 0.90 | 3.00 | 3.00 |  |  |
| Proteose Peptone | 5.00 |  | 1.50 | 15.00 | 10.00 | 0.79 |  |
| Casein Tryptone | / |  | / | 10.00 | / | / |  |
| Tryptone |  |  |  | / |  |  | 10.00 |
| L-Tryptopan | 0.20 |  | 0.06 |  |  |  | / |
| L-Arginine | 1.00 |  | 0.30 |  |  |  |  |
| L-Cysteine | / |  | / | 0.30 |  |  |  |
| L-Cysteine Hydrochlorlde | 0.30 | 0.50 | 0.44 | / | 0.30 |  |  |
| Casein enzymic hydrolysate | / | 15.00 | 10.50 |  | / |  |  |
| Potassium Dihydrogen Phosphate | 2.50 | / | 0.75 | 2.50 | 2.50 |  |  |
| Disodium hydrogen phosphate | / |  | / | | | 0.20 |  |
| Sodium Thioglycolate | 0.30 |  | 0.09 | 0.15 | 0.30 | / |  |
| Sodium chloride | 3.00 |  | 0.90 | 3.00 | 3.00 | 0.39 |  |
| Sodium acetate | / | 5.00 | 3.50 | / | | | |
| Vitamin K1 | 0.0050 | / | 0.0015 |  |  |  |  |
| Hemln | 0.0100 |  | 0.0030 |  |  |  |  |
| Resazurin | / | 0.0025 | 0.0018 |  |  |  |  |

**Table S3. The primers used in qPCR**

| Name | Primer | Sequence |
| --- | --- | --- |
| 16S | Forward | 5’-TCCTACGGGAGGCAGCAGT-3’ |
|  | Reverse | 5’-GACTACCAGGGTATCTAATCCTGTT-3’ |
| Firmicutes | Forward | 5’-TGAAACTYAAAGGAATTGACG-3’ |
|  | Reverse | 5’-ACCATGCACCACCTGTC-3’ |
| Bacteroidetes | Forward | 5’-CRAACAGGATTAGATACCCT-3’ |
|  | Reverse | 5’-GGTAAGGTTCCTCGCGTAT-3’ |
| Verrucomicrobia | Forward | 5’-GAATTCTCGGTGTAGCA-3’ |
|  | Reverse | 5’-GGCATTGTAGTACGTGTGCA-3’ |
| *baiJ* | Forward | 5’-TCAGGACGTGGAGGCGATCCA-3’ |
|  | Reverse | 5’-TACRTGATACTGGTAGCTCCA-3’ |
| *bsh* | Forward | 5’-ATGGGCGGACTAGGATTACC-3’ |
|  | Reverse | 5’-TGCCACTCTCTGTCTGCATC-3’ |
| *bCoA* | Forward | 5’GCIGAICATTTCACITGGAAYWSITGGCAYATG-3’ |
|  | Reverse | 5’-CCTGCCTTTGCAATRTCIACRAANGC-3’ |

| Compound | Q1 (Da) | Q3 (Da) | DP (V) | CE (eV) | EP (V) | CXP (V) |
| --- | --- | --- | --- | --- | --- | --- |
| Aspirin | 179.0 | 137.1 | -21 | -10 | 10 | -16 |
| Salicylic acid | 137.0 | 93.0 | -53 | -19 | 10 | -12 |
| Benzoic acid | 120.8 | 77.0 | -31 | -17 | 10 | -7 |
| Levodopa | 198.1 | 152.2 | -17 | -18 | 10 | 10 |
| Dopamine | 154.2 | 137.3 | 14 | 15 | 10 | 11 |
| Methyldopa | 212.1 | 139.0 | 26 | 25 | 10 | 11 |
| Doxifluridine | 245.0 | 108.0 | -50 | -24 | -10 | -17 |
| 5-Fluorouracil | 129.2 | 42.4 | -50 | -33 | -10 | -17 |
| 5-Chlorouracil | 145.0 | 42.0 | -50 | -16 | -10 | -17 |

**Table S4. MRM transitions and the optimum LC–MS/MS conditions**

**Table S5. The composition of 6 mixed media**

| No. | Method | Media | Percentage of BG (%) | Percentage of mGAM (%) | Percentage of TYG(%) |
| --- | --- | --- | --- | --- | --- |
| 1 | Low proportion mixing | B9T | 90 | / | 10 |
| 2 |  | B8T | 80 | / | 20 |
| 3 |  | B7T | 70 | / | 30 |
| 4 | High proportion mixing | GB | 50 | 50 | 0 |
| 5 |  | BGT | 35 | 35 | 30 |
| 6 |  | B3T | 30 | / | 70 |

**Table S6. PERMANOVA analysis of factors (Bray_curtis)**

| Factor | Df | SumsOfSqs | MeanSqs | F.Models | R^2^ | Pr(>F) |
| --- | --- | --- | --- | --- | --- | --- |
| BG | 1 | 0.972 | 0.972 | 4.821 | 0.211 | 0.001 |
| Residuals | 18 | 3.630 | 0.202 | - | 0.789 | - |
| Total | 19 | 4.602 | - | - | 1 | - |
|  | | | | | | |
| Factor | Df | SumsOfSqs | MeanSqs | F.Models | R^2^ | Pr(>F) |
| GB | 1 | 0.493 | 0.493 | 2.907 | 0.139 | 0.005 |
| Residuals | 18 | 3.051 | 0.170 | - | 0.861 | - |
| Total | 19 | 3.544 | - | - | 1 | - |
|  | | | | | | |
| Factor | Df | SumsOfSqs | MeanSqs | F.Models | R^2^ | Pr(>F) |
| BGT | 1 | 0.965 | 0.965 | 4.922 | 0.215 | 0.001 |
| Residuals | 18 | 3.530 | 0.196 | - | 0.785 | - |
| Total | 19 | 4.495 | - | - | 1 | - |

**Table S7. Amount of sequence data for all the samples (Reads per sample)**

| No. | Sample_info | Seq_num | No. | Sample_info | Seq_num | No. | Sample_info | Seq_num |
| --- | --- | --- | --- | --- | --- | --- | --- | --- |
| 1 | Feces | 53936 | 41 | 24h_BB_2_2 | 62568 | 81 | 6h_BHI_1_2 | 60238 |
| 2 | 6h_mGAM | 30055 | 42 | 24h_BB_3_1 | 60943 | 82 | 6h_BHI_2_1 | 66051 |
| 3 | 12h_mGAM | 36316 | 43 | 24h_BB_3_2 | 72658 | 83 | 6h_BHI_2_2 | 60275 |
| 4 | 24h_mGAM | 43598 | 44 | 6h_BG_1_1 | 71432 | 84 | 6h_BHI_3_1 | 117013 |
| 5 | 6h_BB | 61208 | 45 | 6h_BG_1_2 | 62901 | 85 | 6h_BHI_3_2 | 54790 |
| 6 | 12h_BB | 66913 | 46 | 6h_BG_2_1 | 66917 | 86 | 24h_BHI_1_1 | 55986 |
| 7 | 24h_BB | 53650 | 47 | 6h_BG_2_2 | 48098 | 87 | 24h_BHI_1_2 | 84760 |
| 8 | 6h_BG | 56947 | 48 | 6h_BG_3_1 | 59344 | 88 | 24h_BHI_2_1 | 51681 |
| 9 | 12h_BG | 86915 | 49 | 6h_BG_3_2 | 51253 | 89 | 24h_BHI_2_2 | 87085 |
| 10 | 24h_BG | 54601 | 50 | 24h_BG_1_1 | 55827 | 90 | 24h_BHI_3_1 | 66454 |
| 11 | 6h_mGAM_c | 42338 | 51 | 24h_BG_1_2 | 84262 | 91 | 24h_BHI_3_2 | 68066 |
| 12 | 12h_mGAM_c | 52369 | 52 | 24h_BG_2_1 | 65650 | 92 | 6h_TYG_1_1 | 54108 |
| 13 | 24h_mGAM_c | 50678 | 53 | 24h_BG_2_2 | 59663 | 93 | 6h_TYG_1_2 | 56701 |
| 14 | 6h_GAM | 30841 | 54 | 24h_BG_3_1 | 69900 | 94 | 6h_TYG_2_1 | 56489 |
| 15 | 12h_GAM | 32078 | 55 | 24h_BG_3_2 | 36993 | 95 | 6h_TYG_2_2 | 52087 |
| 16 | 24h_GAM | 47607 | 56 | 6h_mGAM_c_1_1 | 54620 | 96 | 6h_TYG_3_1 | 47332 |
| 17 | Feces_D1 | 66615 | 57 | 6h_mGAM_c_1_2 | 64403 | 97 | 6h_TYG_3_2 | 47964 |
| 18 | Feces_D2 | 56558 | 58 | 6h_mGAM_c_2_1 | 57596 | 98 | 24h_TYG_1_1 | 59514 |
| 19 | Feces_D3 | 68525 | 59 | 6h_mGAM_c_2_2 | 52290 | 99 | 24h_TYG_1_2 | 69808 |
| 20 | 6h_mGAM_1_1 | 58018 | 60 | 6h_mGAM_c_3_1 | 65000 | 100 | 24h_TYG_2_1 | 61571 |
| 21 | 6h_mGAM_1_2 | 62149 | 61 | 6h_mGAM_c_3_2 | 49778 | 101 | 24h_TYG_2_2 | 64140 |
| 22 | 6h_mGAM_2_1 | 62502 | 62 | 24h_mGAM_c_1_1 | 78080 | 102 | 24h_TYG_3_1 | 59694 |
| 23 | 6h_mGAM_2_2 | 55607 | 63 | 24h_mGAM_c_1_2 | 69378 | 103 | 24h_TYG_3_2 | 71113 |
| 24 | 6h_mGAM_3_1 | 57171 | 64 | 24h_mGAM_c_2_1 | 82468 | 104 | Feces_1 | 54781 |
| 25 | 6h_mGAM_3_2 | 68213 | 65 | 24h_mGAM_c_2_2 | 73877 | 105 | Feces_2 | 50959 |
| 26 | 24h_mGAM_1_1 | 49730 | 66 | 24h_mGAM_c_3_1 | 46606 | 106 | Feces_3 | 59392 |
| 27 | 24h_mGAM_1_2 | 64095 | 67 | 24h_mGAM_c_3_2 | 73730 | 107 | mGAM_1_1 | 64076 |
| 28 | 24h_mGAM_2_1 | 57890 | 68 | 6h_GAM_1_1 | 56067 | 108 | mGAM_1_2 | 60201 |
| 29 | 24h_mGAM_2_2 | 51849 | 69 | 6h_GAM_1_2 | 58640 | 109 | mGAM_2_1 | 45465 |
| 30 | 24h_mGAM_3_1 | 71965 | 70 | 6h_GAM_2_1 | 53357 | 110 | mGAM_2_2 | 46478 |
| 31 | 24h_mGAM_3_2 | 70776 | 71 | 6h_GAM_2_2 | 59247 | 111 | mGAM_3_1 | 47205 |
| 32 | 6h_BB_1_1 | 72594 | 72 | 6h_GAM_3_1 | 54248 | 112 | mGAM_3_2 | 51654 |
| 33 | 6h_BB_1_2 | 91402 | 73 | 6h_GAM_3_2 | 52411 | 113 | BG_1_1 | 52734 |
| 34 | 6h_BB_2_1 | 62352 | 74 | 24h_GAM_1_1 | 57917 | 114 | BG_1_2 | 59535 |
| 35 | 6h_BB_2_2 | 51453 | 75 | 24h_GAM_1_2 | 71100 | 115 | BG_2_1 | 49983 |
| 36 | 6h_BB_3_1 | 58094 | 76 | 24h_GAM_2_1 | 74344 | 116 | BG_2_2 | 57651 |
| 37 | 6h_BB_3_2 | 55527 | 77 | 24h_GAM_2_2 | 78075 | 117 | BG_3_1 | 45773 |
| 38 | 24h_BB_1_1 | 61276 | 78 | 24h_GAM_3_1 | 89935 | 118 | BG_3_2 | 44162 |
| 39 | 24h_BB_1_2 | 75306 | 79 | 24h_GAM_3_2 | 66929 | 119 | B7T_1_1 | 62368 |
| 40 | 24h_BB_2_1 | 72576 | 80 | 6h_BHI_1_1 | 72993 | 120 | B7T_1_2 | 58683 |

(**Continued)**

| No. | Sample_info | Seq_num | No. | Sample_info | Seq_num | No. | Sample_info | Seq_num |
| --- | --- | --- | --- | --- | --- | --- | --- | --- |
| 121 | B7T_2_1 | 47689 | 146 | BGT_2_2 | 45432 | 171 | D10_BG | 53394 |
| 122 | B7T_2_2 | 54816 | 147 | BGT_3_1 | 42645 | 172 | D1_GB | 50851 |
| 123 | B7T_3_1 | 61968 | 148 | BGT_3_2 | 61320 | 173 | D2_GB | 51406 |
| 124 | B7T_3_2 | 52129 | 149 | D1_Feces | 51092 | 174 | D3_GB | 53000 |
| 125 | B8T_1_1 | 55560 | 150 | D2_Feces | 47483 | 175 | D4_GB | 54070 |
| 126 | B8T_1_2 | 58227 | 151 | D3_Feces | 46204 | 176 | D5_GB | 53699 |
| 127 | B8T_2_1 | 49157 | 152 | D4_Feces | 42135 | 177 | D6_GB | 44724 |
| 128 | B8T_2_2 | 48867 | 153 | D5_Feces | 46544 | 178 | D7_GB | 43212 |
| 129 | B8T_3_1 | 58797 | 154 | D6_Feces | 50068 | 179 | D8_GB | 53823 |
| 130 | B8T_3_2 | 52455 | 155 | D7_Feces | 51211 | 180 | D9_GB | 48980 |
| 131 | B9T_1_1 | 59821 | 156 | D8_Feces | 41095 | 181 | D10_GB | 43911 |
| 132 | B9T_1_2 | 52809 | 157 | D9_Feces | 45382 | 182 | D1_BGT | 46481 |
| 133 | B9T_2_1 | 49075 | 158 | D10_Feces | 49741 | 183 | D2_BGT | 43835 |
| 134 | B9T_2_2 | 54222 | 159 | D1_B3T | 45810 | 184 | D3_BGT | 45521 |
| 135 | B9T_3_1 | 57388 | 160 | D2_B3T | 40239 | 185 | D4_BGT | 44281 |
| 136 | B9T_3_2 | 57221 | 161 | D3_B3T | 52474 | 186 | D5_BGT | 64127 |
| 137 | GB_1_1 | 63104 | 162 | D1_BG | 55649 | 187 | D6_BGT | 43239 |
| 138 | GB_1_2 | 79579 | 163 | D2_BG | 40791 | 188 | D7_BGT | 39426 |
| 139 | GB_2_1 | 53914 | 164 | D3_BG | 43023 | 189 | D8_BGT | 50059 |
| 140 | GB_2_2 | 47398 | 165 | D4_BG | 46002 | 190 | D9_BGT | 40868 |
| 141 | GB_3_1 | 50781 | 166 | D5_BG | 41976 | 191 | D10_BGT | 43659 |
| 142 | GB_3_2 | 55689 | 167 | D6_BG | 41949 |  | | |
| 143 | BGT_1_1 | 60193 | 168 | D7_BG | 54577 |  |  |  |
| 144 | BGT_1_2 | 68088 | 169 | D8_BG | 41408 |  |  |  |
| 145 | BGT_2_1 | 48370 | 170 | D9_BG | 40255 |  |  |  |

**
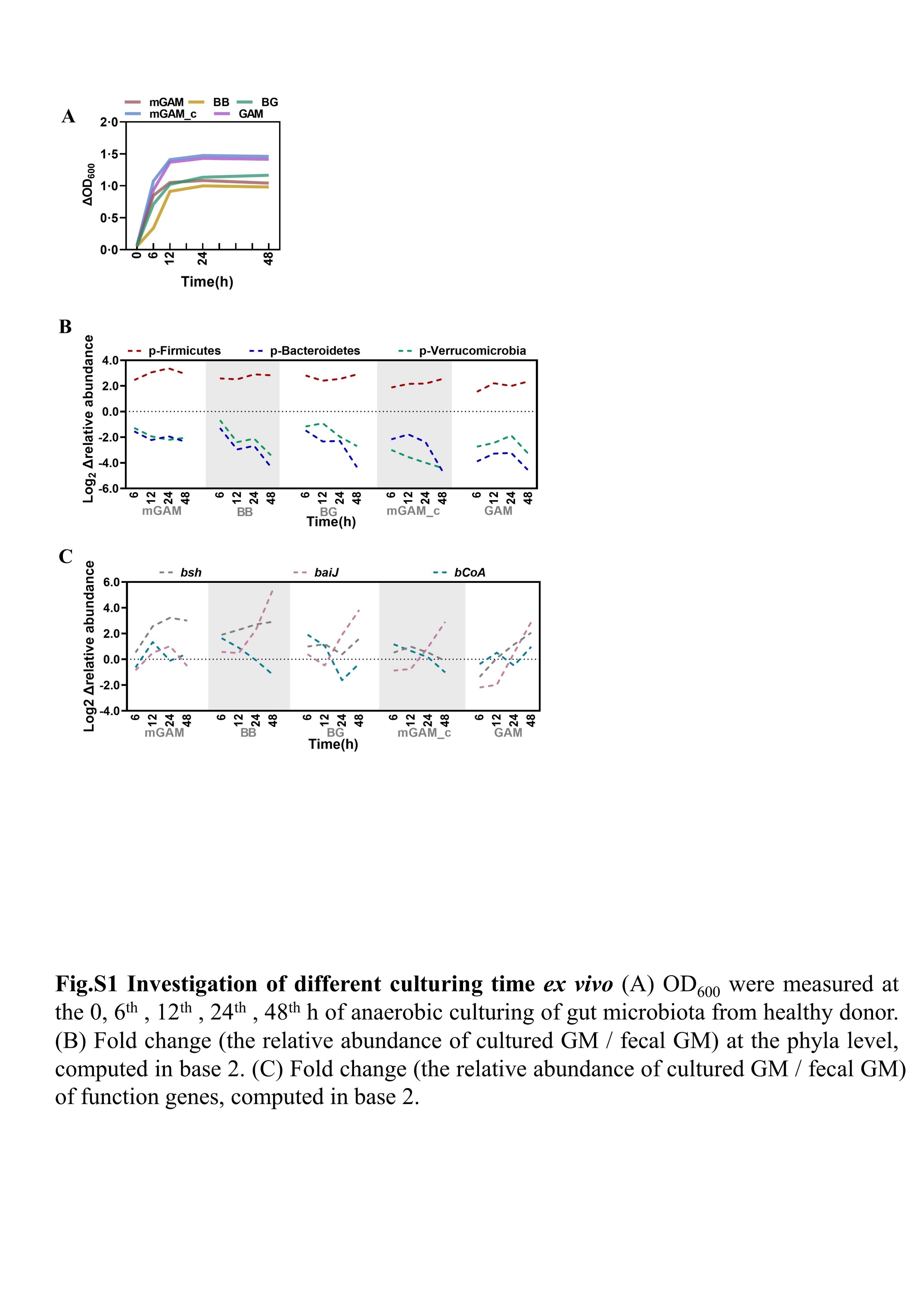
**

**Fig. S1** **Investigation of culture time** **for human-****fecal samples*.*** (A) OD_600_ were measured at the 0, 6^th^ , 12^th^ , 24^th^ , 48^th^ h of anaerobic culturing of gut microbiota from one healthy donor. (B) Fold change (the relative abundance of cultured bacteria/ fecal bacteria) at the phyla level, computed in base 2. (C) Fold change (the relative abundance of cultured bacteria/ fecal bacteria) of function genes, computed in base 2.


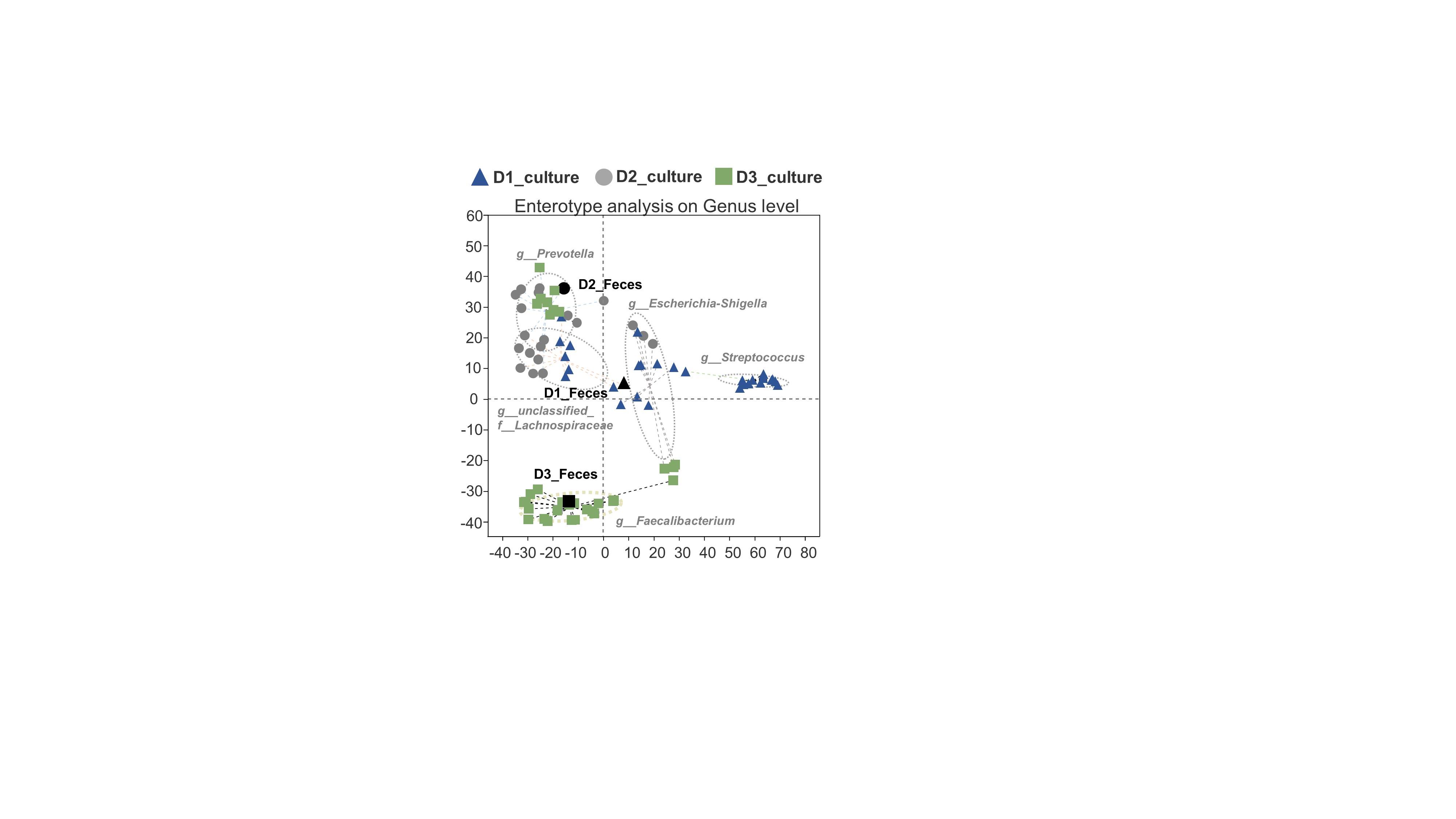
**Fig. S2 Enterotype of fecal samples and their cultures.**


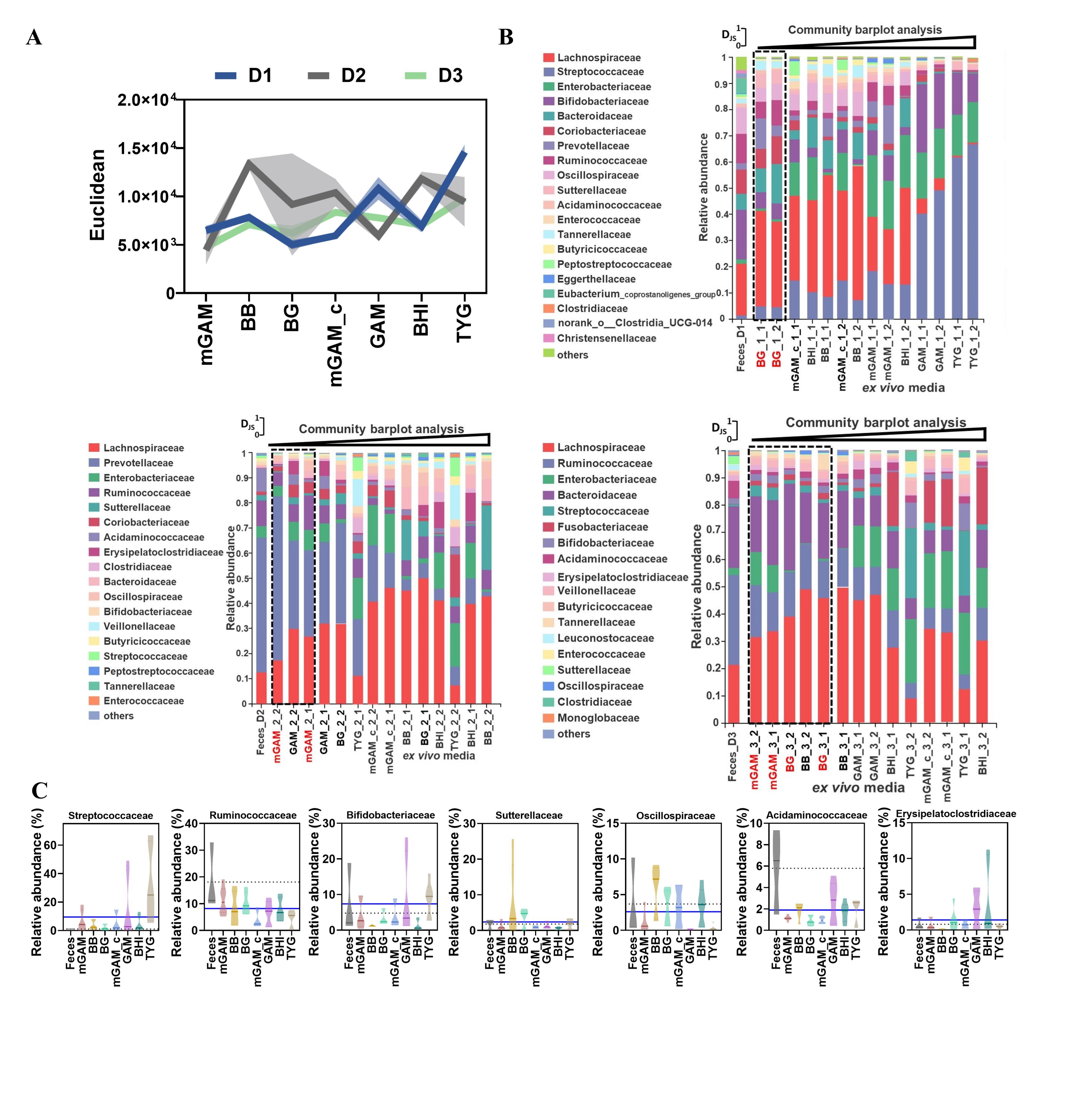
**Fig. S3 Composition of gut microbiota with high abundance.** (A) Euclidean from the original fecal sample at the family level. (B) Family level bacterial composition of the sample, including original fecal sample, as well as its *ex vivo* cultures and repeat (1 and 2), grown anaerobically in 7 different media at incubation time of 24 h. Cultures are ordered according to their D_JS_ from the original fecal sample (upper axes, computed at the family level) (C) Relative abundance of top 4-10 gut microbiota at the family level (dashed line means the abundance of gut microbiota in fresh feces, solid line means the average abundance of gut microbiota in 7 media ).


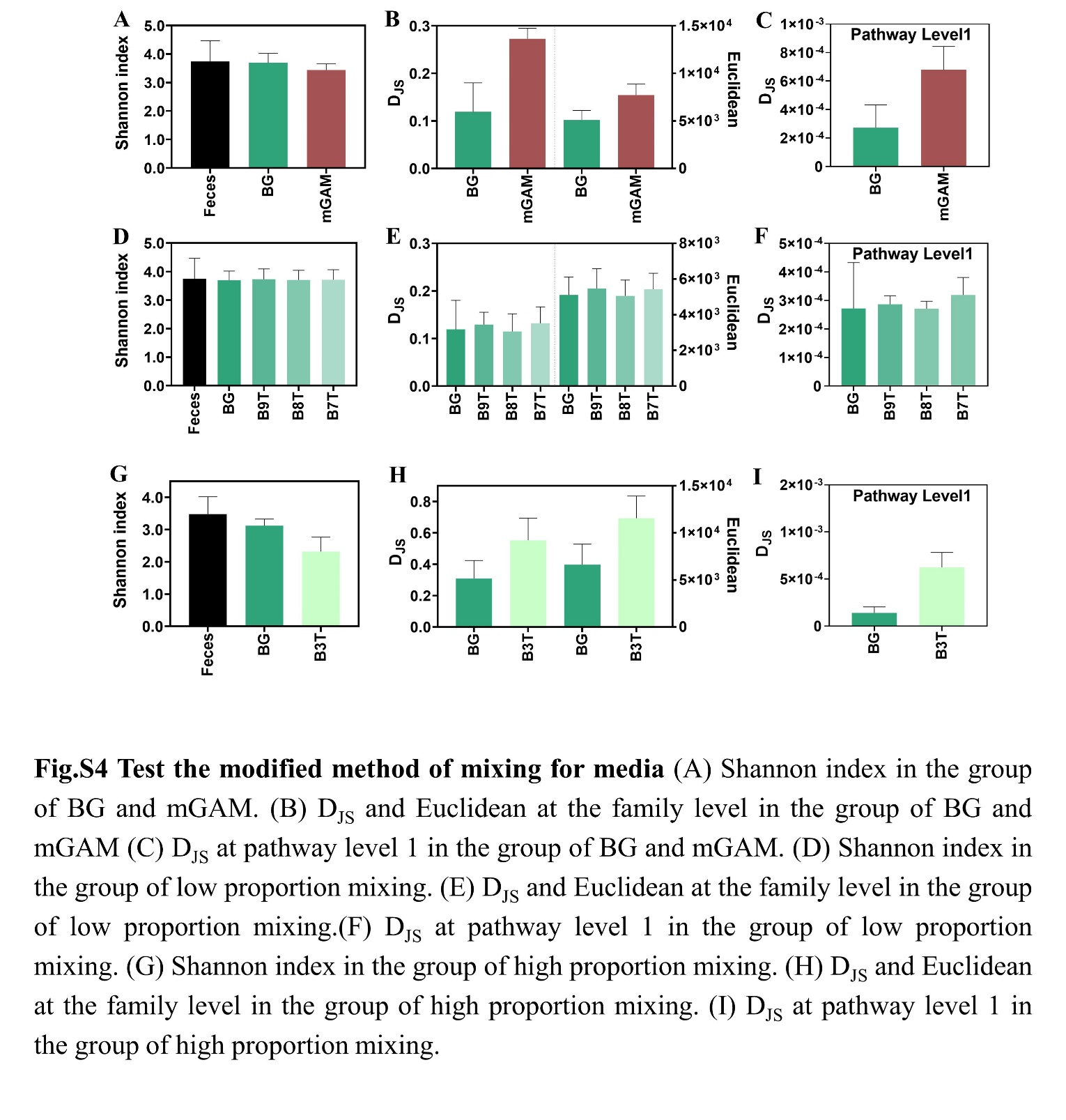
**Fig. S4 The modification of culture media.** (A) Shannon index in the group of BG and mGAM. (B) D_JS_ and Euclidean at the family level in the group of BG and mGAM (C) D_JS_ at pathway level 1 in the group of BG and mGAM. (D) Shannon index in the group of low proportion mixing. (E) D_JS_ and Euclidean at the family level in the group of low proportion mixing.(F) D_JS_ at pathway level 1 in the group of low proportion mixing. (G) Shannon index in the group of high proportion mixing. (H) D_JS_ and Euclidean at the family level in the group of high proportion mixing. (I) D_JS_ at pathway level 1 in the group of high proportion mixing.

**
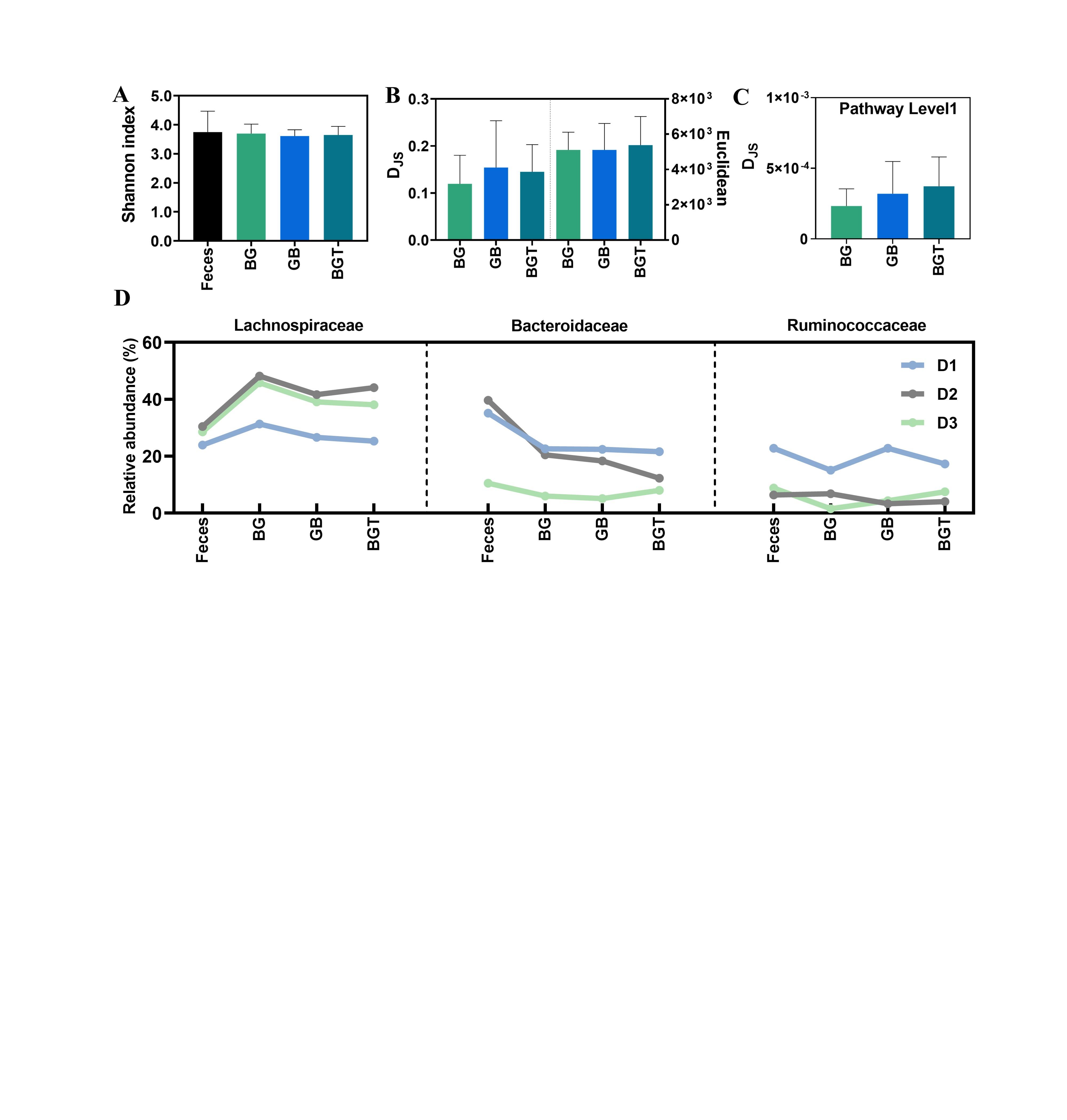
Fig. S5 The optimization of culture media.** (A) Shannon index in the group of high proportion mixing. (B) D_JS_ and Euclidean at the family level in the group of high proportion mixing. (C) D_JS_ at pathway level 1 in the group of high proportion mixing. (D)Abundance of top 3 gut microbiota at the family level.


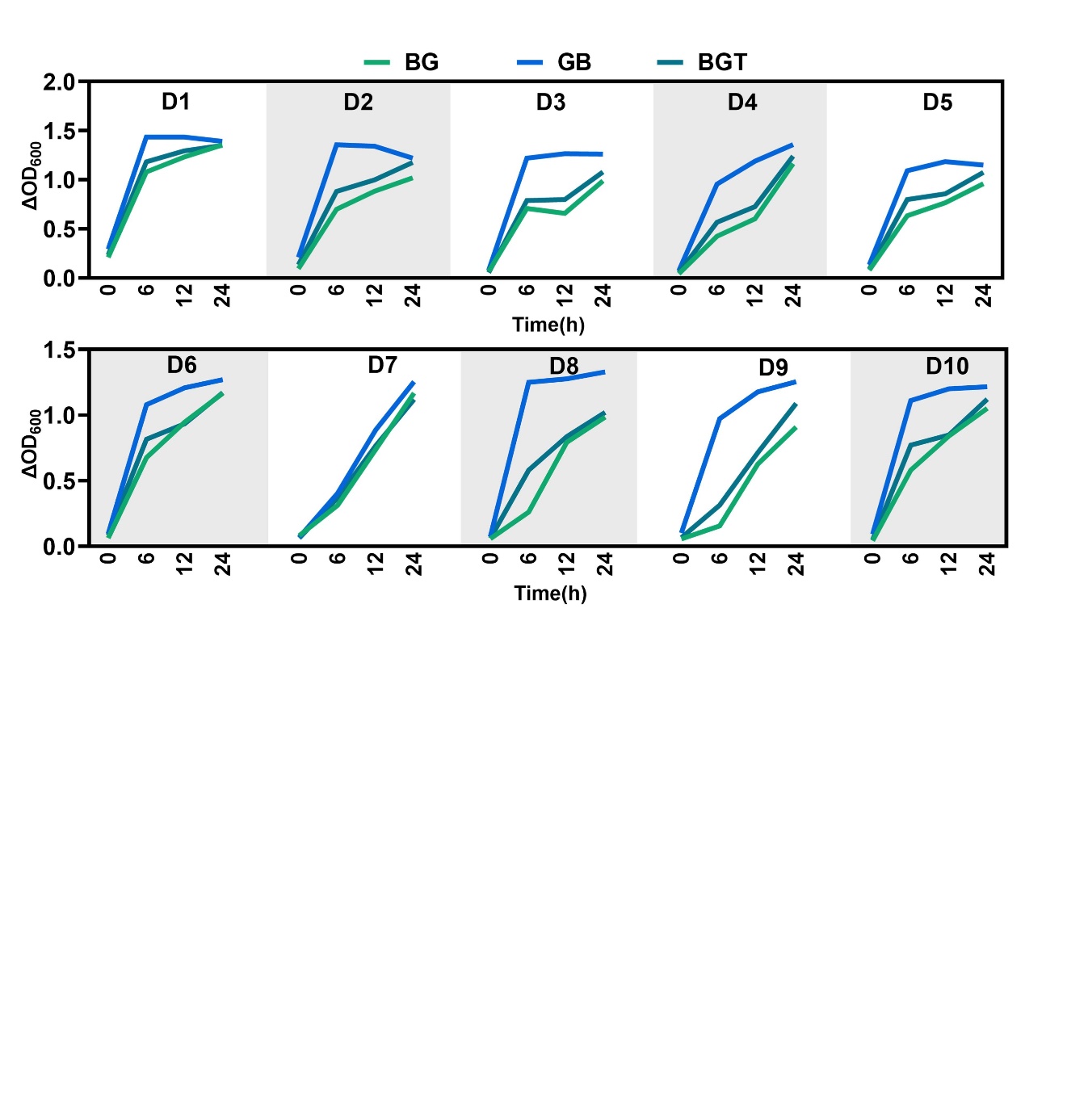
**Fig. S6 Growth condition of optimized culture media.** OD_600_ were measured at the 0, 6^th^ , 12^th^ and 24^th^ h of anaerobic culturing of gut microbiota from D1~D10.


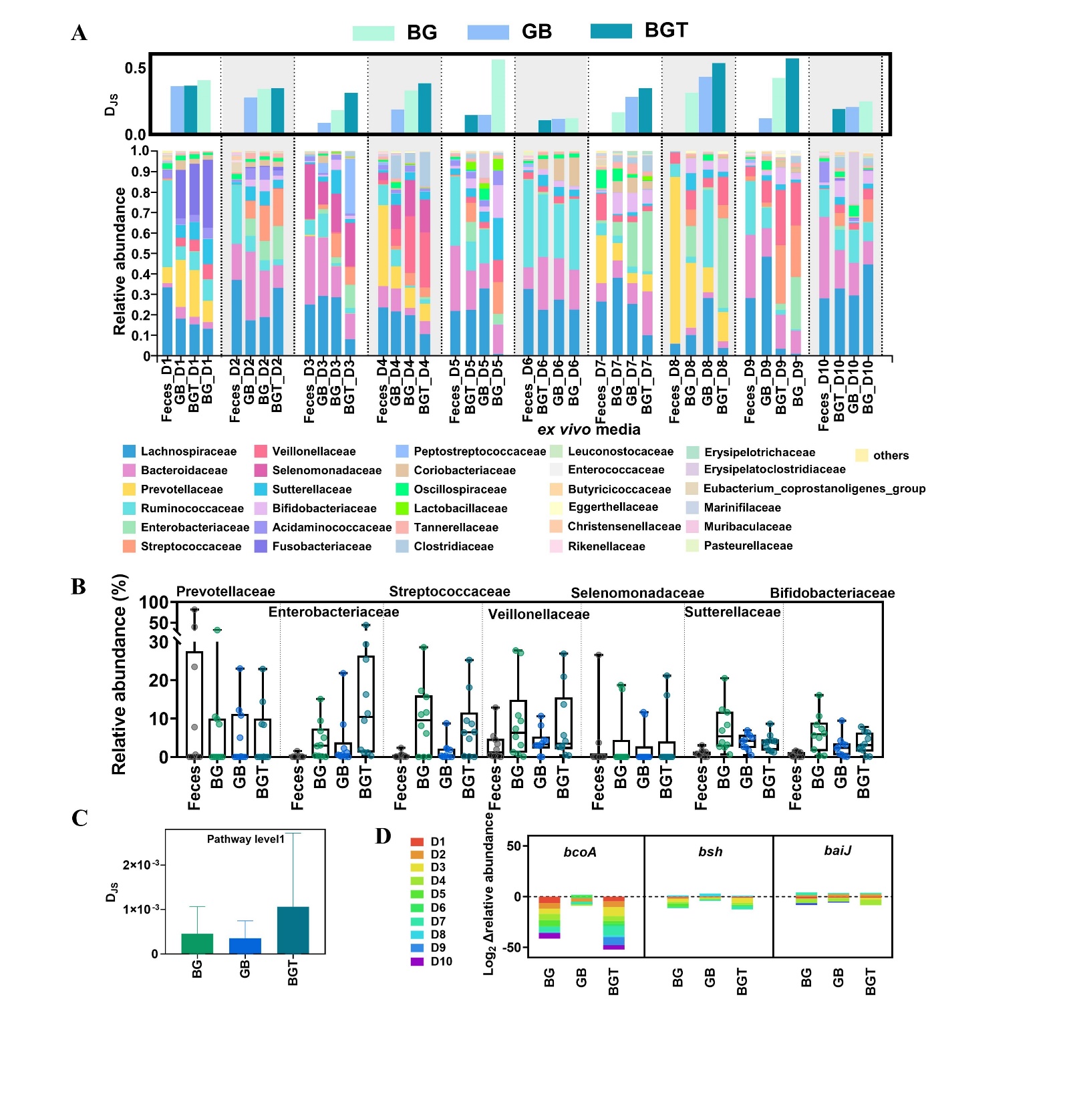
**Fig. S7 Comparing of optimized culture media.** (A) Family level bacterial composition including original 10 fecal samples and its *ex vivo* cultures, grown anaerobically in 3 different mixed media at incubation time of 24 h. Cultures are ordered according to their D_JS_ from the original fecal sample (upper axes, computed at the family level). (B) Relative abundance of top 4-10 gut microbiota at the family level. (C) D_JS_ from the fecal sample, computed at the pathway level 1. (D) Fold change (the relative abundance of cultured bacteria / fecal bacteria) of function genes, computed in base 2.

**
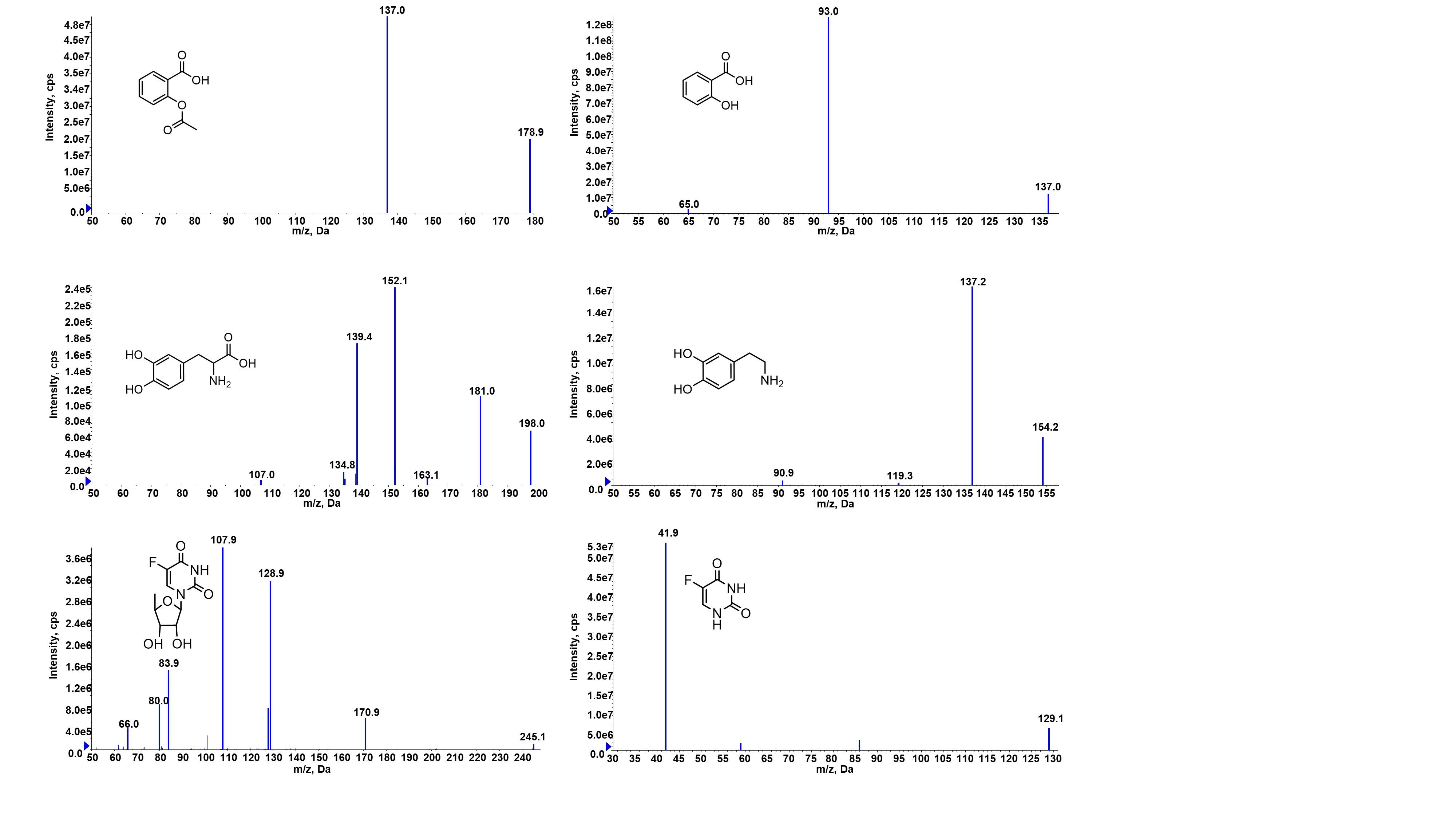
Fig. S8 The mass spectrum.** (A) Parent ion scan of ASA. (B) Parent ion scan of SA. (C) Parent ion scan of L-dopa. (D) Parent ion scan of DA. (E) Parent ion scan of 5’-dFUR. (F) Parent ion scan of 5-FU.
